# Physicians Cannot Testify

**Published:** 1894-06

**Authors:** 


PHYSICIANS CANNOT TESTIFY.—The policy of the law is to make the relation of physician and patient confidential and sacred. Only the patient himself, or, in case of his death, his legal representative, may waive the seal of secrecy and confidence. Thus holds the Supreme Court of Indiana, in the case of Gurley *vs.* Park, decided November 23, 1893. The application given to this doctrine here is to the effect that in a case where there is no legal representative, as administrator or executor, of a deceased person to make waiver, the physician who attended her cannot testify in an action to set aside her will as to her mental condition at the time she made the will, he being present in his professional capacity. The law, this court says, forbids the physician from disclosing what he learns in the sick-room, no matter by what method he acquires his knowledge.—*The Journal of the American Medical Association.*

